# Increases in Nurses' Retired Pay

**Published:** 1920-09-04

**Authors:** 


### Increases in Nurses' Retired Pay.

A NEW Army Order makes provision for increasing the retired pay or pensions of pre-war pensioners whose means, including pension, are under £150 a year if unmarried, or if married, under £200 a year. The pensions subject to increase include nurses' retired pay. If the pensioner lives in the British Isles and has attained the age of sixty (or if a widow of forty), or was retired through physical or mental infirmity, the pension if not over £50 a year may be increased by fifty per cent. Over £50 but not over £100 for the unmarried (£130 for the married), the increase is forty per cent. Over £100 but under £150 the increase is thirty per cent., with proportionate rise for the married. The pension will not in any case exceed £150 a year for the unmarried and £200 for the married. Pre-war pensions already increased by more than the above will not be altered unless the pre-war rate, increased by the above percentages, would give more than the present rate. The increase is to be retrospective as from April 1, 1920. Application should be made to the Secretary, War Office, F 3 Cornwall House, S.E. 1.

